# Vitamin D in Older Adults: The Need to Specify Standard Values with Respect to Cognition

**DOI:** 10.3389/fnagi.2014.00072

**Published:** 2014-04-15

**Authors:** Cédric Annweiler, Olivier Beauchet

**Affiliations:** ^1^Department of Neuroscience, Division of Geriatric Medicine and Memory Clinic, Angers University Hospital, UPRES EA 4638, University of Angers, UNAM, Angers, France; ^2^Department of Medical Biophysics, Schulich School of Medicine and Dentistry, Robarts Research Institute, The University of Western Ontario, London, ON, Canada

**Keywords:** cognition, dementia, older adults, standard, threshold, vitamin D

Besides its classical function of bone metabolism regulation, vitamin D exhibits multiple biological targets mediated by its nuclear hormone receptor, the vitamin D receptor (VDR) (Holick, 2007; Kalueff and Tuohimaa, 2007; Annweiler et al., 2010a, 2011b). Specific actions on target organs such as the central nervous system (CNS) have been described, providing evidence for a neurosteroid action of vitamin D (Kalueff and Tuohimaa, 2007; Annweiler et al., 2010a). Consistently, older adults with lower serum 25-hydroxyvitamin D (25OHD) concentrations exhibit more often and more severe cognitive decline (Annweiler et al., 2009, 2013a,b; Balion et al., 2012; Etgen et al., 2012). At that point, an important issue to be clarified is to determine what concentration of 25OHD is associated with adverse effects in the brain.

## Most Older Adults have Low Levels of Vitamin D

At least one billion people have hypovitaminosis D worldwide (Holick, 2007). Even if all adults can be affected by hypovitaminosis D, older adults have the greatest risk, especially those living in institution (Annweiler et al., 2011b). For illustration, in Europe and the United States, up to 90% of older adults have hypovitaminosis D (Annweiler et al., 2011b). This high prevalence of hypovitaminosis D in the elderly is due to the high frequency of mechanisms leading to hypovitaminosis D, first and foremost the reduction of cutaneous synthesis (decreased exposure to sunlight due to loss of functionality, and decreased capacity of skin synthesis due to the 25% reduction of 7-dehydrocholesterol compared to younger adults) (Holick et al., 1989). Hypovitaminosis D may also result from the alteration of the metabolism of vitamin D (hepatic and kidney failures) (Dusso et al., 2005), inadequate food intake (Hollis and Wagner, 2004), reduced bioavailability (malabsorption syndrome, obesity with sequestration of vitamin D in the fat) (Lo et al., 1985), increased catabolism (regular use of antiepileptics, glucocorticoids, immunosuppressive drugs) (Zhou et al., 2006), and urinary loss of vitamin D (nephrotic syndrome) (Dusso et al., 2005).

The high prevalence of hypovitaminosis D in elderly population is also explained by the choice of the threshold defining hypovitaminosis D, which may vary between 10 and 30 ng/mL.

## Threshold of “Normality” for Serum Vitamin D Concentration

Vitamin D status is usually estimated by measuring serum 25OHD (Holick, 2007; Annweiler et al., 2011b). In general, there are two ways to establish reference values for a biological variable. The first one is based on the use of “population-based reference values,” which comprises measuring a parameter in the reference population size and calculating the reference interval in which there is 95% of the population. In the case of 25OHD, the reference values could not be generalized because they depend on non-modifiable environmental factors (e.g., season, local climate, latitude), modifiable life habits (e.g., clothing, eating habits, sun exposure), and non-modifiable parameters (e.g., ethnicity, skin pigmentation, skin thickness, and age). Moreover, it should be kept in mind that, in clinical practice, the dosage of 25OHD is prescribed to determine if patients need vitamin D supplementation to maintain their health. These values have therefore little interest for the clinicians.

The second method for calculating reference values of 25OHD is to define hypovitaminosis D as 25OHD levels for which there are some adverse health effects. These reference values are called “health-based reference values.” There is an international consensus to use this kind of threshold in the case of 25OHD (Annweiler et al., 2011b). The determination of such a threshold remains yet complex because of the multiplicity of disorders caused by hypovitaminosis D. Thus, historically, “normality” was defined by the avoidance of bone adverse effects. It is classically recognized that there is no rickets or osteomalacia with serum 25OHD above 10 ng/mL (Basha et al., 2000), and no secondary hyperparathyroidism with serum 25OHD above 20 ng/mL (Basha et al., 2000). For now, the threshold at 10 ng/mL (25 nmol/L) remains consensual to define vitamin D deficiency (i.e., severe hypovitaminosis D) (Holick, 2007; Annweiler et al., 2011b), and the threshold at 20 ng/mL (50 nmol/L) is the one used by the World Health Organization to define vitamin D insufficiency (World Health Organization, 2003). However, more recently, it has been elegantly reported that serum 25OHD above 30 ng/mL (75 nmol/L) is required to prevent a number of non-bone effects (Bischoff-Ferrari et al., 2006).

However, it is not clear thus far which of these thresholds matches the eviction of neurological adverse effects of hypovitaminosis D. This would require confronting these three thresholds with the neurological effects that have been described in relation to vitamin D.

## Vitamin D and the Central Nervous System: Preclinical Evidence

Vitamin D is able to enter the cerebrospinal fluid (CSF) and brain by crossing the blood–brain barrier via passive diffusion and additional specific carriers in the cerebral capillaries or the blood–CSF barrier in the plexus choroideus (Holmøy et al., 2009). The concentration of 25OHD in the CSF positively correlates with that in the serum under physiological conditions (Holmøy et al., 2009). *In situ*, vitamin D exerts most of its actions through its nuclear hormone receptor, VDR, expressed in neuronal and glial cells of the CNS, especially the hippocampus, hypothalamus, cortex, and subcortex (Kalueff and Tuohimaa, 2007; Annweiler et al., 2010a). The binding of vitamin D on the VDR triggers neuronal protection against several degenerative processes, including anti-inflammatory action (Moore et al., 2005), antioxidant effect (Ibi et al., 2001), control of calcium homeostasis by regulating the concentration of intracellular calcium in hippocampal neurons (Brewer et al., 2001), anti-atrophic effect by regulating neurotrophic agents (Brown et al., 2003), and attenuation of Aβ42 peptide accumulation (Yu et al., 2011) by stimulating the phagocytosis of Aβ peptide (Masoumi et al., 2009) together with enhancing brain-to-blood Aβ efflux transport at the blood–brain barrier (Ito et al., 2011). Moreover, vitamin D regulates the genetic expression of numerous neurotransmitters in the brain, including acetylcholine, dopamine, serotonin, and γ-aminobutyric acid, notably in the hippocampus (Kalueff and Tuohimaa, 2007). These experimentally described neurosteroid properties of vitamin D may help, in the case of normalized vitamin D status, to address against the decline of brain function in older adults, especially against cognitive decline (Annweiler and Beauchet, 2011).

## Vitamin D and Cognition: Epidemiological Evidence

Most studies on this topic have been conducted in the past decade. It has become clear that older adults with Alzheimer’s disease have lower vitamin D concentrations than others (Balion et al., 2012; Annweiler et al., 2013a). Similarly, hypovitaminosis D is associated with the presence of dementia in cross-sectional studies (Buell et al., 2010). Prospective longitudinal cohorts in older adults have also reported that hypovitaminosis D predicted increased incidence of dementia after 7 years of follow-up (Annweiler et al., 2011a). In contrast, high dietary intake of vitamin D (≥800 UI/day reduced the incidence of Alzheimer’s disease after 7 years (Annweiler et al., 2012a). The relationship between vitamin D and dementia is highlighted during the advanced stages of the disease (Annweiler et al., 2011), but also from the prodromal stage (mild cognitive impairment, MCI) (Annweiler et al., 2012b) even though this minor neurocognitive disorder does not diminish the functional autonomy. Finally, the relationship between vitamin D and cognition was also found in people without dementia, with a linear relationship (Annweiler et al., 2009). The lower the concentration of vitamin D, the more impaired the cognitive performance (Oudshoorn et al., 2008). This direct association was found with both the global cognitive performance (Etgen et al., 2012), the memory, and especially with the executive functions (Annweiler et al., 2013b). In other words, vitamin D is associated with cognitive performance in older adults even before the onset of dementia. It is thus crucial to appreciate, in older adults with or without dementia, what level of vitamin D is associated with impaired cognitive scores.

## Analytical Study of the Threshold of Serum 25OHD Concentration Related to Cognitive Disorders

Table 1 summarizes the characteristics of the studies that have explored the association of cognitive scores with hypovitaminosis D defined as serum 25OHD concentration less than either 10, 20, or 30 ng/mL.

**Table 1:** **Summary of the observational studies examining the association between cognitive scale scores and hypovitaminosis D using a threshold at 10, 20, or 30 ng/mL**.

Reference	Settings	Population	Subgroups of serum 25-hydroxyvitamin D	Association with cognitive scale scores?
**THRESHOLD 10 ng/mL**				
Wilkins et al. (2006)	Location: Washington, DC, USA (38.9°N)	Community-dwellers	<10 Versus ≥20 ng/mL	Yes, with global cognitive performance
		*N* = 80, 62.5% women	
		Mean age: 74.8 ± 7.7 years	
		22.5% Black	
		Specificity: half with mild dementia, and half without dementia	
Aung et al. (2006); *CREST study*	Location: Harris County, TX, USA (29.8°N)	Community-dwellers	<10 Versus ≥10 ng/mL	No, with global cognitive performance No, with executive functions
		*N* = 44, 63.6% women		
		Mean age: 76.1 years	
		38.6% White	
		Specificity: self-neglecting older adults referred by the adult protective services	
Buell et al. (2009); *NAME study*	Location: Boston, MA, USA (42.2°N)	Community-dwellers	<10 Versus 10–20 ng/mL	No, with global cognitive performance
		*N* = 1080, 75.9% women	
		Mean age: 75.0 ± 8.5 years	
		34.9% Black	
		Specificity: older adults receiving home health services	
				No, with information processing speed
				Yes, with mental shifting
				Yes, with information updating
Annweiler et al. (2010b); *EPIDOS study*	Location: France (Toulouse, 43.4°N; Montpellier, 43.6°N; Lyon, 45.5°N; Paris 48.5°N; Amiens, 49.9°N)	Community-dwellers	<10 Versus ≥10 ng/mL	Yes, with global cognitive performance
		*N* = 752, 100% women	
		Mean age: 81.2 ± 3.5 years	
		Specificity: only older women	
**THRESHOLD 20 ng/mL**				
Wilkins et al. (2006)	Location: Washington, DC, USA (38.9°N)	Community-dwellers	10–19.9 Versus ≥20 ng/mL	Yes, with global cognitive performance
		*N* = 80, 62.5% women	
		Mean age: 74.8 ± 7.7 years	
		22.5% Black	
		Specificity: half with mild dementia, and half without dementia	
Aung et al. (2006); *CREST study*	Location: Harris County, TX, USA (29.8°N)	Community-dwellers	<20 Versus ≥20 ng/mL	No, with global cognitive performance No, with executive functions
		*N* = 44, 63.6% women		
		Mean age: 76.1 years	
		Specificity: self-neglecting older adults referred by the adult protective services	
Buell et al. (2009); *NAME study*	Location: Boston, MA, USA (42.2°N)	Community-dwellers *N* = 1080, 75.9% women Mean age: 75.0 ± 8.5 years 34.9% Black	10–20 Versus >20 ng/mL	Yes, with global cognitive performance
				No, with information processing speed
				No, with mental shifting
				No, with information updating
		Specificity: older adults receiving home health services	
Wilkins et al. (2009)	Location: Washington, DC, USA (38.9°N)	Community-dwellers	<20 Versus ≥20 ng/mL	No, with global cognitive performance
		*N* = 60	
		Mean age: 75.0 ± 8.2 years	
		50.0% Black	
		Specificity: either cognitively normal or mildly impaired cognition, without vitamin D supplementation	
Hansen et al. (2011)	Location: Bergen, Norway (60.4°N)	*N* = 25, 100% men	<20 Versus ≥20 ng/mL	No, with information processing speed Yes, with information updating
		Mean age: 34.6 ± 9.4 years		
		Specificity: only middle-aged men incarcerated in a Norwegian prison	
Menant et al. (2012); *Memory and Aging Study*	Location: Sydney, NSW, Australia (33.5°S)	Community-dwellers *N* = 463, 53.4% women Mean age: 78.0 ± 4.6 years	≤20 Versus >20 ng/mL	No, with global cognitive performance
				No, with information processing speed
				Yes, with mental shifting
**THRESHOLD 30 ng/mL**				
Llewellyn et al. (2011); *NHANES III*	Location: USA (25.5°N – 47.3°N)	Community-dwellers *N* = 3325, 55.2% women Mean age: 73.7 ± 10.9 years 7.6% Black Specificity: only older adults	<10 Versus ≥30 ng/mL	Yes, with global cognitive performance
				Yes, with information processing speed
				Yes, with information updating
				Yes, with episodic memory
Llewellyn et al. (2010); *InCHIANTI study*	Location: Italy (Greve in Chianti, 43.6°N; Bagno a Ripoli, 43.8°N)	Community-dwellers	<10 Versus ≥30 ng/mL	Yes, with global cognitive performance Yes, with information processing speed Yes, with mental shifting
		*N* = 858, 49.4% women		
		Mean age at baseline: 73.9 years		
		Caucasian	
		Specificity: older adults from a geographically confined area	
Slinin et al. (2012); *SOF study*	Location: USA (Baltimore, MD, 39.3°N; Pittsburgh, PA, 40.4°N; Portland, OR, 45.6°N; Minneapolis, MN, 46.9°N)	Community-dwellers	<10 Versus ≥30 ng/mL	Yes, with global cognitive performance Yes, with mental shifting
		*N* = 5692, 100% women		
		Mean age at baseline: 76.6 ± 4.7 years	
		Caucasian	
		Specificity: only older women	

As illustrated, the results were mixed, with some studies having found an association between hypovitaminosis D and cognitive disorders (Wilkins et al., 2006; Buell et al., 2009; Annweiler et al., 2010b; Llewellyn et al., 2010, 2011; Hansen et al., 2011; Menant et al., 2012; Slinin et al., 2012), while others reported no association (Aung et al., 2006; Buell et al., 2009; Wilkins et al., 2009; Hansen et al., 2011; Menant et al., 2012). Of note, it was primarily the threshold at 10 ng/mL that was associated with cognition, whereas this was not the case with the threshold at 20 ng/mL. Additionally, the convincing results with the threshold at 30 ng/mL were constantly found in comparison with concentrations lower than 10 ng/mL, which reinforces the clinical value of the latter threshold with respect to cognition.

Highlighting that the threshold of 25OHD at 10 ng/mL is linked to cognition makes sense. Indeed, the brain is able to withstand degenerative lesions for a long time before expressing an objectified cognitive decline (Jack et al., 2010). In other words, occurrence of cognitive disorders means that the brain is already the seat of advanced neuronal damages. Precisely, since hypovitaminosis D occurs gradually, presenting with 25OHD concentration lower than 10 ng/mL means that hypovitaminosis D is chronic (Annweiler et al., 2011b), and has probably led to brain dysfunction for a long time. In line with this, it has already been shown that the lower the 25OHD concentration, the more severe the chronic diseases (Beauchet et al., 2012).

## Implications for Practice and Research

The existing body of evidence provides proof that the threshold of 25OHD associated with cognitive status is around 10 ng/mL, the people with 25OHD concentration <10 ng/mL having a greater risk of cognitive disorders than those with 25OHD >10 ng/mL, and an even greater risk compared to those with 25OHD >30 ng/mL. The implications for practice and research are manifold. First, this finding supports the idea that chronic hypovitaminosis D is a risk factor for cognitive disorders, and may partially explain the onset of dementia among older adults. Second, it means that older adults with cognitive disorders likely have very low 25OHD concentrations and, thus, should receive vitamin D supplementation in clinical routine to prevent both skeletal and non-skeletal adverse consequences of hypovitaminosis D. Third, this finding is interesting for the conduct of future clinical trials testing the cognitive efficacy of vitamin D supplements. Since it is useless to give vitamin D supplements to people who already have a satisfactory rate (Annweiler and Beauchet, 2013), it appears appropriate to include in future trials only older participants who have 25OHD concentrations <10 ng/mL. Conversely, including participants with higher initial rates might mask the cognitive effects of supplementation (if any). What is more, it is legitimate to propose a supplement plan designed to achieve a final 25OHD concentration >30 ng/mL.

## Conclusion

In conclusion, older adults commonly have low serum vitamin D concentrations. For clinicians, it is useful to determine the level of vitamin D required to prevent the development of diseases. Regarding cognition, existing literature provides evidence that the threshold of 25OHD associated with cognitive disorders is somewhere around 10 ng/mL. Unfortunately no study has tested yet the three classical 25OHD thresholds simultaneously in relation to cognition. Prospective multi-center population-based cohort studies are desirable to address this issue specifically with a satisfactory level of evidence.

## Author Contributions

All authors meet all of the following criteria: (1) contributing to the conception and design, or analyzing and interpreting data; (2) drafting the article or revising it critically for important intellectual content; and (3) approving the final version to be published.

## Conflict of Interest Statement

The authors declare that the research was conducted in the absence of any commercial or financial relationships that could be construed as a potential conflict of interest.
